# Mr. Saumariez, on Generation, and the Principle of Life

**Published:** 1799-10

**Authors:** Richard Saumariez

**Affiliations:** Newington, Surry


### *To the Editors of the Medical and Phyſical Journal.*

GENTLEMEN,

I SEND you theſe few obſervations on the ſubject of Generation, and the principle of Life; being a brief analyſis of what I have publiſhed more at large on the ſame ſubject. If you think them deſerving of notice, you are at liberty to inſert them in the Medical and Phyſical Journal.

I am, &c.

Your's very ſincerely,

NEWINGTON, SURRY,

*Sept.* 14*th*, 1799.

RICHARD SAUMAREZ.

The

The Medical Journal of the laſt month contains a paper on *Animal Impregnation*, evidently written for the purpoſe of confirming two opinions, which have lately been broached and entertained on the ſubject. The one is, that the palpable application of ſemen to the ovarium does not take place;—and ſecondly, that the exiſtence of corpora lutea conſtitutes the true teſt of animal impregnation.

In order to prove the error of the firſt opinion, I ſhall trace the various modes by which we know fœcundation is accompliſhed in ſome orders of the more ſimple ſyſtems, as it will enable us to underſtand the manner in which it is effected in thoſe of a more complicated ſtructure. Such is the regularity which vegetables, and the lower orders of animals, diſplay in the actions they perform, that we are neceſſarily led to conclude, that thoſe actions are governed by fixed and general principles, which they cannot either ſuppreſs or prevent; there is an appointed period of growth for the different organs in general of each, and an appointed ſeaſon for the evolutions of particular organs, and when the diſpoſition for their reſpective actions begins and ends. The alteration which vegetables periodically undergo, from a torpid to an active ſtate, until fructification is accompliſhed, is obvious to every obſerver. In many vegetables the propagation of the ſpecies is not confined to one, but extends to ſeveral different modes, viz. by branches and buds, by ſuckers, by leaves, and by ſeed; when the propagation of the ſpecies is the conſequence of ſeed, the organ by which it is produced is found to be reſident, for the moſt part either in the ſame branch, or encloſed within the ſame calyx. There ariſes either directly from the ſummit, or from the ſides of the germen or ſeed-bud, an erect column called piſtillum, the baſe of which has received the appellation of ſtyle, and which is terminated by the ſtigma, or crown of the piſtil, and is generally found with a downy covering of a moiſt quality; it is this organ which LINNÆUS ſuppoſed conſtitutes the female part of generation. External to the piſtil we find the ſtamina to be ſituated; the baſe immediately ariſes from the plant, and proceeding in a thread-like form is called *filamentum*, which is terminated by the *anthera;* the anthera generally conſiſts of two cavities, which contain a fine farinaceous powder, analogous to the *ſemen maſculinum*, called pollen; theſe cavities ultimately burſt, ſo that the pollen which is ſhed from the anthera, or ſummit of the ſtamen is received by the ſtigma, or ſummit of the piſtillum, ſo that an union of both takes place. Theſe are the means which vegetables employ to celebrate their connubium, or marriage, and the mode by which it is conſummated. It appears to me impoſſible to do away the croud of facts which prove the power which the pollen contains, and the faculty it has of imparting the character of the ſyſtem from whence it is produced, to the female ſyſtem by which which it is received; the various hybrid productions that are the consequent result establish the fact beyond controversy.

If we proceed from vegetables to the lower order of animals, we find that although the mode of propagation may be limited with respect to them, that it is far superior to what the higher classes possess. The sexes are also not only particularly distinguished, but there is evidently sexual intercourse between them: in them the mode of propagation is limited to one, requiring the union of two subjects before it can be accomplished. The first order of these animals is called hermaphrodite, when both sexes, male and female, are found existing in the same system—the snail, the slug, the leech, &c. belong to this class. Although hermaphrodite animals possess both sexes, it does not appear that the different sexes of the same system ever copulate together; the union of two separate systems is necessary to call forth the combined actions of the four sexual organs at one and the same time.

When we go to examine the generating organs of different animals, we shall find that the evolution they undergo at particular seasons is great and striking. The evolution of those organs is less evident in the higher than it is found in the lower classes—less evident in the human species than in quadrupeds—in quadrupeds than it is in birds, in the amphibia, in fish, or in vegetables. The direct evidence we possess that the semen of the male is applied in a palpable form to the ova of the female in the latter systems, lead us to make an analogical conclusion, that it takes place also in the former, although the manner is different, arising from the difference in the nature of their organization; I shall therefore proceed to examine the mode by which fœcundation in them is accomplished.

The organs of generation in fish consist of two testes, and two ovaria: the system that possesses the one is called the male fish; the other is distinguished by the appellation of female. If either are examined in the winter season, during their torpid state, both these organs are found flaccid and empty; on the contrary, when viewed in the spring and summer, when the evolution in the system in general has taken place, these parts in particular appear distended and full. The testes, which are distinguished by the whiteness of their colour and softness of their texture, have received the appellation of roe, and are then full of a white fluid called semen. The female organs are called ovaria, known by the name of hard roe, and are completely full of ova.

When these parts have attained the full perfection of their evolution, they are expelled from each system; the semen of the male unites with the ova of the female, and fœcundation ensues, without sexual intercourse between both, both. It is with a view of accomplishing this end that fish in general go in shoals—that particular classes of fish have particular latitudes for their habitation, and particular situations to which they resort at particular seasons, in order that the spawn which they shed may immediately combine together, an union takes place between the semen and the ova, without any intercourse between the parents, and fœcundation ensues to an extent far surpassing any example we witness of the most complicated frame.

In the amphibia, and birds, the same enlargement in the fœcundating organs is equally apparent. The animals that belong to the former class consist especially of the frog, the toad, the turtle, the lizard, and all of the snake kind. I shall take the frog as an example, because the changes the male and female undergo are more striking than in any other. We have constant opportunities of beholding the palpable application of the male semen to the female ova.

The male frog has a testis situated in the loins, having an excretory duct called *vas deferens*, communicating with a *vesicula seminalis*, which finally terminates at the *anus*. The female frog has a number of small ova, attached to a membrane, which is connected to the loins somewhat similar to the male testis. There is an oviduct terminating in an uterus, to which it is attached. The ovarium and the testes are remarkably small during the autumnal and winter months; but as the winter cold departs, and the vernal warmth succeeds, the testes and the ova become gradually developed, and ultimately assume a considerable size; so that when these animals are examined in the spring, the appearance they display is totally different from what they manifested in the winter. Instead of being thin and flat, languid and torpid, they are sound, lively, and active. The male is plump and fat, the female distended, and swelled to a considerable size: and finally, instead of subsisting in a state of separation and divorce, they are found embracing each other, and consummating their union. Animals that are in this state are said to have the *œstrum* upon them. The male climbs upon the back of the female, passes his arms over her shoulders, and adheres to the surface of her body in such a manner that the vas deferens, which terminates at the anus, is placed exactly above the vagina; this is the condition in which they are found, and which they preserve for a fortnight, until the final cause of their union is accomplished: the final cause of their union in the female consists in the expulsion of the ova which the ovarium contains—in the male it consists in the discharge of the semen from the testis, through the medium of the vas deferens, upon the ova, so that they become sprinkled by it in proportion as they are repelled, constituting the mode by which fœcundation is accomplished.

The

The mode of propagation in this prolific fyftem, although very fimple, is even more complicated than it is in fifh. In fifh there exifts a feparation between the male and female, but an union only between the femen and ova from without; in frogs there is an union between the male and female in general, before fœcundation can be accomplifhed.

The higher fpecies of the amphibious clafs are all of the fnake kind: in them we find a confiderable degree of difference which fubfifts;—inftead of fœcundation taking place without the ufe of fexual organs, fœcundation can be accomplifhed by means of fexual organs alone. The male has two *teftes*, with two *vafa deferentia*, which terminate not at the anus, as in the frog, but with two diftinct penes, or male fexual organs, the furface of which are covered over with numerous papillæ. The female has two fets of ovaria, which extend from the middle of the animal's body to its pofterior extremity, containing an abundant quantity of ova; there are two Fallopian tubes, or oviducts, which receive the ova from the ovaria, and convey them to the uterus, from whence they are expelled. Although the mode of fœcundation is different in thefe higher fyftems, the end is evidently the fame as in the inferior; the femen, inftead of uniting with the ova out of the body, is conveyed within by the agency of the fexual organs of the male, through the medium of the Fallopian tubes, to the ovaria of the female, in order that it may unite with the ova which are fufficiently evolved, that fœcundation may be accomplifhed. That the Fallopian tubes poffefs the power of conveying the femen to the ovaria, is evidently proved from the ftrong and active periftaltic motion they difplay, and which appears evidently defigned in the firft inftance to convey the femen to the fimbriæ; while the fimbriæ, which before only covered a fmall portion of the ovaria, are gradually expanding themfelves, fo as to grafp and completely enclofe the ovaria. It is by the wonderful reciprocity of action at this time in thefe various parts, that the femen is applied to the furface of the ovaria, and the ova which have evolved and enlarged become fœcundated by the union of the femen with them.*

On examining a doe rabbit, which I killed two hours and a half after fhe had been admitted to the male, independant of the inward vafcularity of the Fallopian tubes, and ftrong periftaltic motion—of the progreffive attachment of the fimbriæ to the ovaria—and of the protruding condition of feveral ova in them—I do declare, that I difcovered a fluid in colour and confiftency exactly fimilar to æther, and which fpread itfelf as æther is wont to do, when rubbed

* Having given in detail all the experiments that illuftrate the fubject of animal fœcundation in the Syftem of Phyfiology which I lately publifhed, I muft refer the reader to the work itfelf.

rubbed between the fingers, supported by that portion of the expanded fimbriæ which had not yet grasped the ovarium. I firmly believe that this was the fluid destined to impregnate the ova. I do not, however, wish to dwell too long on one solitary fact, when the analogy is so strong and so general, that it cannot be resisted. The union of the semen to the ova is proved directly in the whole inferior order of animated beings I have examined—in the amphibia—in fish—and in vegetables; why then should it be denied to the higher classes?—For no other reason than the mere supposition that "it is absorbed from the vagina, and conveyed to the general system, where, by its peculiar stimulus it produces the changes which happen *after* impregnation in the uterus—its appendages and the breasts perfecting what the stimulus of coition had begun." This is the mere *ipse dixit* of your correspondent, unsupported by proof, refuted by analogy, arising from his ignorance of the true end for which the act of coition is designed.

In proportion as we ascend in the chain of animated existence, we find a considerable abatement in the effects which œstrum alone produces; the power and disposition to action in the generating organs progressively diminishes, requiring causes of a more active nature than we behold in the lower orders. The power which the female of oviparous animals possesses of evolving the ova she contains when the season for fœcundation is present, does not extend to the animals of an higher class, by virtue of that power alone; a necessity absolutely exists that in them sexual union should take place, not only for the proper secretion of semen, but for the evolution of the ova. The excitement which the ovaria sustain during, an d in consequence of that act, constitutes the only means by which the ova can evolve, and become separated from the capsules in which they are inclosed—in the lower orders, a total separation of the semen and of the ova ensues, although no sexual unions have happened has taken place.

[To be resumed in our next Number.]

---

